# Use of RNA-seq data to identify and validate RT-qPCR reference genes for studying the tomato-*Pseudomonas* pathosystem

**DOI:** 10.1038/srep44905

**Published:** 2017-03-20

**Authors:** Marina A. Pombo, Yi Zheng, Zhangjun Fei, Gregory B. Martin, Hernan G. Rosli

**Affiliations:** 1 Instituto de Fisiología Vegetal, INFIVE, Universidad Nacional de La Plata, CONICET, La Plata, Buenos Aires, Argentina; 2 Boyce Thompson Institute for Plant Research, 533 Tower Road, Ithaca, NY 14853, USA; 3 USDA-ARS Robert W. Holley Center for Agriculture and Health, Ithaca, NY 14853, USA; 4 Section of Plant Pathology and Plant-Microbe Biology, School of Integrative Plant Science, Cornell University, Ithaca, NY 14853, USA

## Abstract

The agronomical relevant tomato-*Pseudomonas syringae* pv. *tomato* pathosystem is widely used to explore and understand the underlying mechanisms of the plant immune response. Transcript abundance estimation, mainly through reverse transcription-quantitative PCR (RT-qPCR), is a common approach employed to investigate the possible role of a candidate gene in certain biological process under study. The accuracy of this technique relies heavily on the selection of adequate reference genes. Initially, genes derived from other techniques (such as Northern blots) were used as reference genes in RT-qPCR experiments, but recent studies in different systems suggest that many of these genes are not stably expressed. The development of high throughput transcriptomic techniques, such as RNA-seq, provides an opportunity for the identification of transcriptionally stable genes that can be adopted as novel and robust reference genes. Here we take advantage of a large set of RNA-seq data originating from tomato leaves infiltrated with different immunity inducers and bacterial strains. We assessed and validated 9 genes that are much more stable than two traditional reference genes. Specifically, *ARD2* and *VIN3* were the most stably expressed genes and consequently we propose they be adopted for RT-qPCR experiments involving this pathosystem.

Gene expression quantification is an important and widely used technique that allows analyzing the state of different cellular processes in specific conditions. Nowadays, reverse transcription-quantitative PCR (RT-qPCR) is the tool most frequently used to determine the mRNA levels in different biological systems^1^. Because of its sensitivity, accuracy and rapid execution, it is considered the most important mid-throughput gene expression analysis technology^2^. However, there are several critical steps during the execution of RT-qPCR experiments that affect the accuracy and interpretation of the results, such us the quality of the mRNA, amplification efficiency and the choice of reliable internal controls referred to as reference genes^2^,^3^.

Normalization of the sample expression with reference genes is used to account for the technical variation produced during the processing of the samples. A reference gene is an internal control that should have minimal or no variation of its expression in the analyzed conditions^2^,^4^. Traditionally, few reference genes are used in plants such as beta-tubulin-4 (*TUB4*), glyceraldehyde-3-phosphate dehydrogenase (*GADPH*), 18S ribosomal RNA (*18S RNA*), polyubiquitin (*UBQ*), actin (*ACT*), elongation factor 1 alpha (*EF1α*)^4^. Because of their relatively high expression levels in all kinds of cells or tissues, these genes were initially selected as reference genes for qualitative (Northern blot) and semi-quantitative (RT-PCR) approaches and have been widely adopted for RT-qPCR experiments^5^. However, several recent studies indicate that these traditional reference genes are not very stably expressed in different experimental conditions and have encouraged the systematic selection and validation of better RT-qPCR reference genes previous to performing expression level measurements^2^,^5^,^6^,^7^,^8^.

Plants detect and react to pathogens using a two-layer defense mechanism. Pattern-triggered immunity (PTI) is activated after the detection of microbe/pathogen-associated molecular patterns (MAMPs or PAMPs) by membrane receptors called pattern-recognition receptors (PRRs)^9^,^10^,^11^. Some bacterial pathogens use a type III secretion system to introduce effector (virulence) proteins into the cell cytoplasm to undermine PTI^12^. During evolution, some plants developed the ability to recognize the activity of these effectors and activate a second layer of immunity named effector-triggered immunity (ETI)^12^,^13^,^14^. Large changes in gene expression occur during the development of both immune responses^15^,^16^,^17^,^18^.

The interaction between tomato (*Solanum lycopersicum*) and the causal agent of bacterial speck disease, *Pseudomonas syringae* pv. *tomato (Pst*), is considered a model for the study of molecular mechanisms leading to plant defense responses. The perception of bacterial flagellin by tomato has been well characterized. This protein contains two MAMPs that are detected by tomato: flg22 and flgII-28, recognized by FLS2 and FLS3 receptors, respectively^19^,^20^,^21^,^22^. It has been reported that the primary PTI elicitors from *Pst* in tomato are the flagellin-derived MAMPs and this perception results in extensive transcriptional changes^17^. Around 30 effectors are introduced into plant cells by *Pst* strain DC3000^23^. Among them, AvrPto and AvrPtoB are early-acting effectors that suppress PTI by interfering with PRR-mediated signaling and thereby promote bacterial virulence^17^,^24^,^25^. In some tomato lines, members of the Pto kinase family detect and interact with AvrPto and AvrPtoB effectors and jointly with the nucleotide binding-leucine rich repeat (NB-LRR) protein Prf activate ETI^26^,^27^,^28^,^29^. Changes in tomato gene expression that occur during Pto/Prf-mediated ETI were previously studied^18^,^30^.

Recently, several tomato genes have been evaluated and identified as the most suitable RT-qPCR reference genes in different experimental conditions. For example, there are now reference genes available for tomato fruit development^31^, tomato seeds under different conditions^32^ and MicroTom-*Rg1* genotype fruit^33^. Similar studies have been conducted in tomatoes under abiotic stresses^34^ and biotic interactions, such as host responses to viruses^35^,^36^,^37^ and *Xanthomonas campestris* pv. *vesicatoria (Xcv*)^38^. Theses studies support the idea that there is not a single reference gene that can be used for a given species, and consequently these need to be evaluated and selected for each particular expression study.

In most of these tomato publications, the selection of the candidate reference genes was based on genes previously used for the same species in different experimental conditions or for other phylogenetically related plants. Alternatively, in some cases, the authors used transcriptional expression data generated by microarray analysis to identify novel and more stably expressed genes when compared to traditionally employed reference genes^38^. In the past years, RNA-seq has emerged as a powerful high-throughput technology used for transcriptome analysis in different organisms and treatments^39^,^40^,^41^,^42^,^43^. In spite of being a technique used for many years, RNA-seq data has been used in the plant research field for the selection and validation of new and more robust RT-qPCR reference genes only in grape, soybean and *Lycoris*^44^,^45^,^46^.

Previously, we used an RNA-seq approach for the analysis of PTI activation in tomato and the subsequent inhibition of this response by *Pst* effectors AvrPto and AvrPtoB^17^. Additionally, we identified genes that are differentially expressed specifically during activation of PTI or ETI in tomato^18^. Here, we have taken advantage of the large set of RNA-seq data mentioned above in addition to newly generated data that complements the publicly available set, for the selection of 9 candidate genes with the lowest variation within a total of 37 different treatments/time-points and their biological replicates. We then performed RT-qPCR experiments using tomato leaf tissue infiltrated with different *Pseudomonas* species and mutants to study their behavior upon activation of plant defense. Validation of these genes was performed using three different tools (geNorm, NormFinder and Bestkeeper), and compared with two traditional housekeeping genes and the most stably expressed gene identified during the analysis of tomato infection with *Xcv*^38^. Our results identified a set of novel reference genes that are transcriptionally more stable than the traditional ones and consequently we propose their use in experiments involving tomato-*Pseudomonas* pathosystem.

## Results

### Selection of tomato genes with stable expression using RNA-seq data

In order to identify genes whose expression has a low variation across different treatments, we took advantage of the RNA-seq expression data previously published^17^,^18^ and newly generated additional data. Treatments are described in Supplementary Table S1 and include different bacterial strains and MAMP infiltrations, along with mock treatments and untreated leaf tissue at different time-points. In all these experiments, the leaf tissue was collected at 30 min, 4 and 6 h after infiltration (hai) to investigate early changes in host gene expression (Supplementary Table S1).

In order to rank the predicted 34,725 tomato genes (ITAG 2.4^47^) based on their transcript level stability, we calculated the variation coefficient (VC) using all the RPKMs (reads per kilobase of transcript per million mapped reads) determined in each experimental condition (37 different conditions, Supplementary Table S2) using the biological replicate information individually. This accounted for 110 total values. The lower the VC is, the more stable the expression of the gene is across all the conditions. In this way we selected 9 genes with the lowest VC for analysis, none of which had been used previously as tomato reference genes in RT-qPCR assays (Supplementary Table S2). For this set of genes, VC ranged from 12.2% to 14.4%. We also selected a gene named *PHD* (Solyc06g051420, VC 31.5%) previously identified and validated reference gene in tomato-*Xanthomonas campestris* pv. *vesicatoria (Xcv*) pathosystem^38^ and two traditional plant reference genes *EF1α* (Solyc06g005060, VC 41.6%) and *GADPH* (Solyc04g009030, VC 52.9%). Gene expression variability across all the RNA-seq treatments of the selected genes was globally analyzed in a log_2_(RPKM) box plot graph (Supplementary Fig. S1). Relative box and whisker sizes indicated low gene expression stability of *PHD*, *GAPDH* and *EF1α.*

### Expression profiles of candidate reference genes showed good amplification efficiencies and primer specificities

We performed RT-qPCR using cDNA dilutions (1:5, 1:10, 1:100, 1:1000). Amplification efficiency E was measured as 10^−1/slope^ and expressed in percentage (Supplementary Table S3). All the primers designed in this work showed high amplification E values ranging from 89% to 117%. Another important aspect to be evaluated is the specificity of the amplification. To achieve this, we performed melting curves for all the pair of primers used and in all cases observed a single peak corresponding to a single amplification product (Supplementary Fig. S2).

### Cycle amplification values (Cq) indicate a wide range of expression levels among the selected tomato reference genes

We designed an experiment aimed at evaluating the performance of our set of genes under different plant immune responses. Therefore, in order to activate PTI we infiltrated tomato Rio Grande (RG)-PtoR leaves with *Pseudomonas fluorescens* 55 (*Pf*)^48^ and 10 mM MgCl_2_ as a mock treatment. Also, we infiltrated the RG-PtoR tomato leaves with *Pst* DC3000^49^ to activate PTI and ETI and the double mutant *Pst* DC3000 Δ*avrPto* Δ*avrPtoB*^50^ to induce the development of bacterial speck disease (Table 1). We collected leaf tissue from 3 biological replicates at early time points, 6 and 12 hai, and monitored the development of symptoms in these plants at later time points to confirm the activation of the expected plant responses.

**Figure 1:**
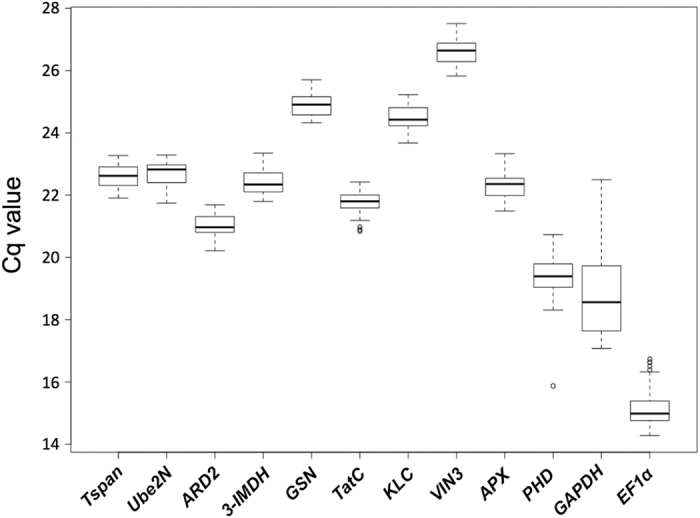
Cycle quantification (Cq) values of selected genes. Box and whisker plot graph showing Cq values of each selected gene in all the samples analyzed (n = 24). Black lines and boxes represent the medians and 25/75 percentiles, respectively. Whisker caps represent the minimum and maximum values. Ο, indicates outliers.

Average Cq values, ranged from 14.9 (*EF1α*) to 26.6 (*VIN3*) (Fig. 1) indicating most of the genes (except for *EF1α*) have a Cq value that is within the recommended values for a RT-qPCR reference gene (higher than 15 and lower than 30)^4^. Moreover, *GADPH* expression levels were the most variable, with minimum and maximum values of 17.1 and 22.5, respectively. This represents a difference of 5.4 Cq between them. Importantly, this difference (max Cq - min Cq) was much smaller, ranging between 1.4 and 1.8, for the genes identified in this work.

### Different algorithms indicate *ARD2* and *VIN3* are the most stable reference genes

**Figure 2:**
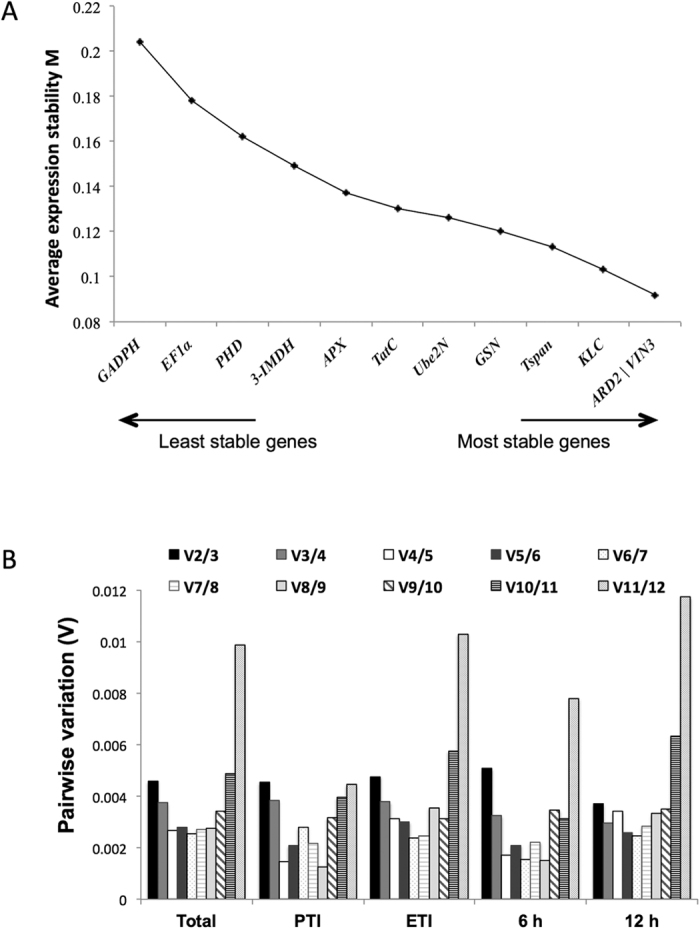
geNorm analysis of selected reference genes in the tomato-*Pseudomonas* pathosystem. (**A**) Tomato reference genes were ranked based on expression stability calculated by geNorm. M values represent the average pairwise variation of the gene compared with all other control genes. (**B**) Pairwise variation (Vn/Vn + 1) for determination of the optimal number of reference genes. The pairwise variation was calculated considering all the samples together (Total), mock and *Pf* inoculations (PTI), *Pst* DC3000 and *Pst* DC3000 Δ*avrPto* Δ*avrPotB* (ETI), samples taken at 6 hpi (6 h) or samples taken at 12 hpi (12 h).

To determine which of the selected genes had the most stable expression levels in our system, we analyzed RT-qPCR data with three different tools to estimate gene expression stability. We first determined the average expression stability value M using geNorm software^51^. This program calculates the pairwise variation of each reference gene with all other genes analyzed under the same experimental conditions. In this way, the lower the M value, the more stable the gene is. All the analyzed genes, presented M values lower than the usually proposed cutoff value of M ≤ 0.5. The highest variability was observed for *GADPH* (M = 0.204), *EF1α* (M = 0.178) and *PHD* (M = 0.162) (Fig. 2A). The algorithm also selects an optimal pair of reference genes and in our case the most stable ones were *ARD2* and *VIN3* with an M value of 0.092.

We determined the pairwise variation (V) of a normalization factor (NF) calculated by introducing reference genes one by one, starting from the two least variable until the whole set was included. With this approach, the optimal number of reference genes to be used can be estimated. We analyzed our data as a whole, only including PTI activation (*Pf* 55 and mock), only including ETI activation (*Pst* DC3000 and *Pst* DC3000 Δ*avrPto* Δ*avrPtoB*), only including 4 hai or only including 12 hai (Fig. 2B). Regardless of the plant response activation or time-point, V2/3 value is considerably smaller than the proposed cut-off (<0.15^51^), suggesting that using only the two most stable reference genes (*ARD2* and *VIN3*) is sufficient for normalization. The addition of *GAPDH* (V11/12), the least stable gene, resulted in a particularly large increase of the variation parameter V when analyzing subsets that include ETI induction (Fig. 2B). To look into this phenomenon, we analyzed the individual Cq values for each gene in all conditions (Supplementary Fig. S3). We observed a clear Cq value increase of *GADPH* when ETI is activated (infiltration with *Pst* DC3000) at 6 and 12 hai, suggesting down regulation of the corresponding transcript. This result indicates *GADPH* is not a suitable reference for experiments involving ETI activation.

**Figure 3:**
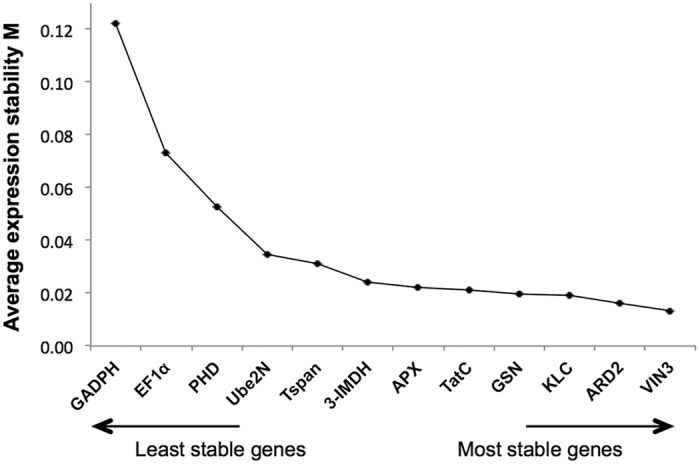
Expression stability of selected reference genes in the tomato-*Pseudomonas* pathosystem using NormFinder. Tomato reference genes were ranked based on expression stability calculated by NormFinder. The analysis was performed using expression data from all biological replicates and treatments (n = 24).

To further investigate the gene expression stability of the selected genes in our experiments, we analyzed our data with NormFinder^52^. This algorithm also calculates an M index, but taking into account the intragroup (within each sample/treatment) and the intergroup variation (within different groups of samples/treatments). This analysis revealed similar results than geNorm (Fig. 3). The most suitable reference genes derived from NormFinder analysis were *VIN3*, *ARD2* and *KLC* with M values of 0.013, 0.016 and 0.019, respectively. On the other hand, *GADPH*, *EF1α*, and *PHD* were among the least stable genes.

The other tool we used to study candidate gene stability, BestKeeper, allows the analysis of up to 10 reference genes^53^. For this reason, we included in the analysis the top 7 most stable candidate genes based on NormFinder analysis, *PHD* and the 2 classical reference genes. This tool performs the analysis in two steps. First, it estimates different statistical parameters that allow determining if a gene has an acceptable overall variation to be considered a reference gene (SD [±Cq] < 1 and SD [±x-fold] < 2). All the studied genes, except *GADPH*, passed this filter (Table 2). Then, a matrix of pairwise comparisons and coefficient of correlation (*r*) calculation are performed to obtain a BestKeeper index. The *r* value obtained from the comparison of each gene with this index allows establishing a ranking of reference gene suitability. Higher gene expression stability is associated to *r* values closer to 1. Our results indicate that *APX*, followed by *ARD2* and *VIN3* posses the higher correlation coefficients being the most stable genes of the 10 analyzed (Table 2). Again, *EF1α* and *PHD* were ranked as among the least stable genes with *r* values of 0.061 and 0.125, respectively.

Although the results obtained in this study were largely consistent when comparing the outputs of the statistical programs used, a few discrepancies were observed. It has been proposed that geNorm, NormFinder and BestKeeper tools tend to generate distinct ranking orders of reference genes because they are based on different algorithms^35^. Therefore, it is recommended to consider them as complementary statistical methods and analyze results globally. Thus, we calculated the arithmetical mean of the ranking value obtained for each gene using all three algorithms^35^. As expected, *ARD2* and *VIN3* were rated as the most stable with a mean value of 1.67 (Table 3).

### Validation of the selected genes confirmed their suitability as reference genes

To validate the selection of reference genes, we measured the expression of a PTI- and an ETI-specific gene that were previously reported^18^. As recommended^51^, we estimated the relative expression using the normalization factor NF calculated as the geometric mean of Cq values obtained for *ARD2* and *VIN3*, the most stable reference genes. Alternatively, we selected the worst condition, which is using the least stable gene (*GADPH*) as the only reference.

**Figure 4:**
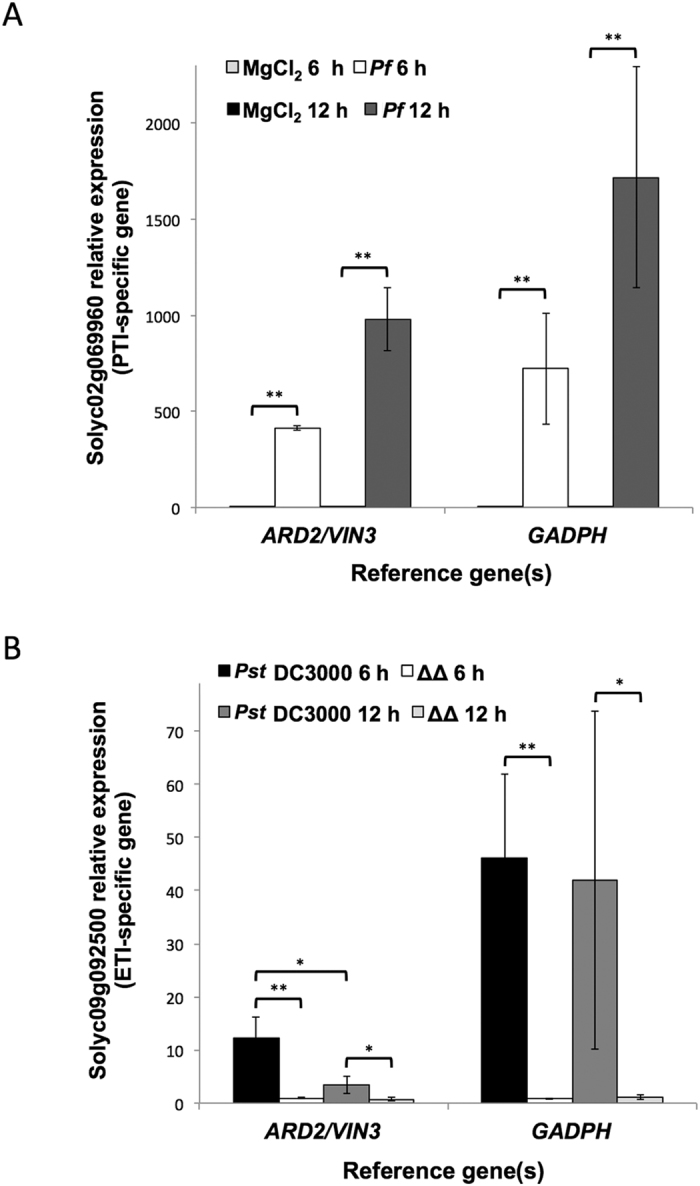
Relative expression of immunity specific reporter genes analyzed using different reference genes. RT-qPCR of (**A**) PTI-reporter gene (Solyc02g069960) at two time points (6 and 12 hai) with mock (10 mM MgCl_2_) or 10^8^ cfu/ml of *Pseudomonas fluorescens* 55 (*Pf*) and (**B**) ETI-reporter gene (Solyc09g092500) at two time points (6 and 12 hai) with 5 × 10^6^ cfu/ml of *Pseudomonas syringae* pv. *tomato* DC3000 (*Pst* DC3000) and *Pst* DC3000 Δ*avrPto* Δ*avrPtoB* (ΔΔ) strains. In both cases, the geometric mean of the two best (*ARD2*/*VIN3*) or the worst (*GADPH*) reference genes were used for normalization of the data. Bars represent the mean of three biological replicates and three technical replicates with their corresponding standard deviation. ** or * indicate significant differences using Student *t-test* with *p*-values < 0.01 or <0.05, respectively.

In the case of the PTI-specific marker (Solyc02g069960), we saw the expected increase of gene expression in the samples infiltrated with *P. fluorescens* 55 (Fig. 4A) at both 6 and 12 hai, regardless of the reference gene used. Although the trend is the same, normalization with an unsuitable reference gene such as *GADPH*, not only increased gene expression levels, but also resulted in larger standard deviation values.

As anticipated, we observed an increase in ETI marker gene (Solyc09g092500) expression in RG-PtoR tomato leaves infiltrated with *Pst* DC3000 compared with *Pst* DC3000 Δ*avrPto* Δ*avrPtoB* (Fig. 4B). Nevertheless, the expression pattern was quite different if the data was analyzed using *ARD2*/*VIN3* or *GAPDH* as reference genes (Fig. 4B). As observed for the PTI reporter gene, the activation of the ETI marker gene was over-estimated. Additionally, the gene expression reduction between 6 and 12 h previously reported^18^, could not be observed when using *GAPDH* normalization. Again, the combined use of *ARD2*/*VIN3* leads to a drastic reduction in standard deviation values.

**Figure 5:**
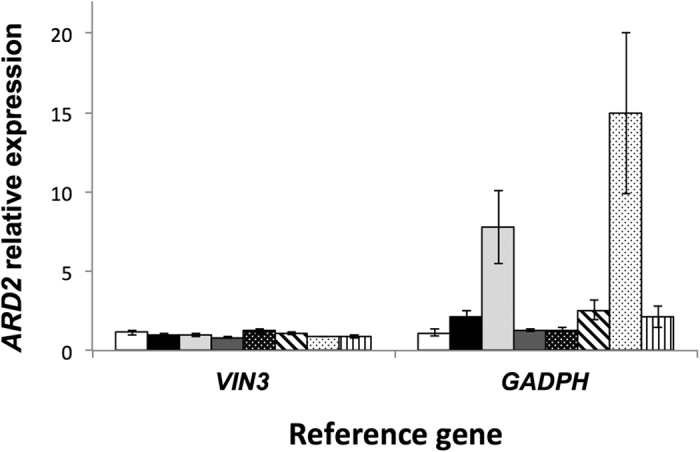
Analysis of *ARD2* relative expression using two different reference genes. RT-qPCR of *ARD2* expression in tomato leaves infiltrated with mock (10 mM MgCl_2_), 10^8^ cfu/ml of *Pseudomonas fluorescens* 55 (*Pf*), 5 × 10^6^ cfu/ml of *Pseudomonas syringae* pv. *tomato (Pst*) DC3000 (DC3000) and *Pst* DC3000 Δ*avrPto* Δ*avrPtoB* (ΔΔ) strains. Samples were taken at two time points (6 and 12 hai). *VIN3* (best) or *GADPH* (worst) reference genes have been used for normalization of the data. Bars represent the mean of three biological replicates and technical replicates with their corresponding standard deviation.

To further investigate the influence of using a non-stably expressed gene as reference in RT-qPCR experiments, we analyzed *ARD2* expression using *VIN3* or *GADPH* as reference gene (Fig. 5). In this analysis, we show that *ARD2* is expressed with remarkably small variation across the different experimental conditions when using *VIN3* as a reference. However, when we normalized the data with *GADPH* the same gene falsely increased its expression upon plant defense activation. This was more evident in the case of the ETI-inducing treatment (*Pst* DC3000), which can be explained by the noticeable down-regulation of *GADPH* gene expression we observed in the samples infiltrated with *Pst* DC3000 (Supplementary Fig. S3). Therefore, our results support the importance of the selection and validation of accurate reference genes RT-qPCR to avoid misinterpretation of the expression data.

## Discussion

RT-qPCR is a powerful technique for gene expression detection and quantification, but the accuracy and reliability of the results highly depend on appropriate data normalization^4^. In this sense, several reports in different plant species like *Arabidopsis*^54^, soybean^45^, rice^55^, cotton^56^ among others, have supported the importance of identifying stably expressed genes for each species, tissue, treatment or condition to be analyzed.

As a new approach for the tomato-*Pseudomonas* pathosystem, we have taken advantage of previously published RNA-seq data^17^,^18^ for the selection of stably expressed genes. In both studies, different infiltrations were performed in tomato leaves aiming at analyzing transcriptional changes during PTI and ETI activation, and the influence of bacterial effectors on plant defenses. To complete this transcriptomic set of information, we performed new experiments that include untreated tomato plants and infiltrations with additional MAMPs (flagellin and non-flagellin derived) and bacterial strains and mutants. Together these data formed a robust set of gene expression information (37 different treatments/time points with an average of 3 biological replicates generated in independent experiments, Supplementary Table S2) that allowed us to select genes with low variation coefficients in the tomato-*Pseudomonas* pathosystem. To our knowledge, this study uses the largest set of RNA-seq data to date to identify reference genes.

For validation of our set of reference genes using RT-qPCR we selected treatments that involve a strong activation of transcriptomic changes. For example challenges with different bacterial strains at the concentration and time-point used lead to approximately 2,800 and 5,700 genes differentially expressed for PTI^17^ and ETI^18^, respectively. With this in mind, we are confident that we tested our candidate genes under rigorous conditions for steady gene expression. In addition, it is worth noting that these infiltration experiments were performed independently from the RNA-seq ones, adding even greater strength to our results. We employed three widely used tools for the evaluation of gene expression stability such as geNorm^51^, NormFinder^52^ and Bestkeeper^53^, to test our reference gene candidates. Our analysis suggests that all 9 selected genes from the RNA-seq data are more stable than the ones commonly used in the literature.

Based on our results, we strongly recommend the use of *ARD2* and *VIN3* as the most suitable reference genes for gene expression studies in tomato leaf interactions with *Pseudomonas* (Table 3). Pairwise variation analysis that geNorm program performs, established that the use of these two genes is sufficient to obtain consistent results. These two selected genes were consistently grouped among the most stable ones, and the traditional *GADPH* and *EF1α* were included within the least stable group.

The interaction of tomato with *Xanthomonas campestris* pv. *vesicatoria (Xcv*) is another commonly used model system for studying plant-pathogen biology^57^. In a recent work, two genes (*PHD* and *LSM7*) were recommended, based on microarray data identification, for normalization in tomato gene expression assays of plants infected with *Xcv*^38^. The authors found that *GADPH* was particularly not a suitable reference gene for this pathosystem, due to its down regulation upon *Xcv* challenge. In our case we observed this same effect in *GADPH* transcript levels when AvrPto/AvrPtoB-mediated ETI response was activated (Supplementary Fig. S3). Contrastingly, *GADPH* was ranked as one of the most stable candidates analyzed for the pathosystem *Actinidia deliciosa*-*Pseudomonas syringae* pv. *actinidiae*^58^. To test how a tomato-*Xcv* suitable reference gene would perform in our system and compare its stability to our set of candidates, we included *PHD*^38^. In spite of *PHD* performing better than the traditional reference genes used in our analysis, all the genes we selected based on RNA-seq data were found to be more stably expressed in the tomato-*Pseudomonas* pathosystem. These findings support the idea that reference genes need to be identified and tested for each specific system.

To put our selected reference genes to test, we performed RT-qPCR experiments to investigate transcript levels of PTI- and ETI-specific reporter genes previously identified^18^. The comparison of the gene expression values obtained when normalizing the data with the combination *ARD2*/*VIN3* or *GADPH* was highly discordant in terms of estimated transcript levels and standard deviations (Fig. 5). This result also emphasizes the importance of the selection of appropriate reference genes to avoid misinterpretation of experiments and further confirmed that *GADPH* is not a good reference gene for expression studies in the tomato-*Pseudomonas* interaction.

In conclusion, by using a large RNA-seq data set we were able to identify and validate highly stable RT-qPCR reference genes. We recommend the use of these genes for gene expression analyses of tomato tissues infected with the bacteria *Pseudomonas syringae* or related experiments. Our results strongly support the importance of taking advantage of high-throughput transcriptomic data currently available for the selection of proper reference genes in RT-qPCR experiments.

## Methods

### Bacterial strains and growth conditions

Bacterial strains used were: *Pseudomonas fluorescens* 55^48^, *Pseudomonas syringae* pv. *tomato (Pst*) DC3000^49^ and *Pst* DC3000 Δ*avrPto* Δ*avrPtoB*^50^, *Pst* DC3000 Δ*hopQ1-1*^59^, *Pst* DC3000 Δ*hrcQ-U* and *Pst* DC3000 Δ28E^24^. All of them were grown on King’s B medium at 30 °C. Antibiotics used were: ampicillin (100 μg/ml) for *Pseudomonas fluorescens* 55 and rifampicin (10 μg/ml) for *Pst* DC3000 and mutants.

### Plant material and treatments

For RNA-Seq analysis, 4-week old Rio Grande (RG-PtoR, *prf3* and *prf19*)^60^ tomato plants were vacuum or syringe infiltrated with bacterial suspensions and MAMPs, sampled at 30 min., 4 h or 6 h, frozen in liquid N_2_ and stored at −80 °C until processed. Additionally, non-treated tissue was processed in the same way. A detail of the treatments performed in this work along with those from previous works^17^,^18^ is shown in Supplementary Table S1. For RT-qPCR analysis, 4-week old resistant Rio Grande-PtoR plants were syringe-infiltrated with a suspension of 10^8^ cfu/ml *P. fluorescens* 55, 5 × 10^6^ cfu/ml *Pst* DC3000, 5 × 10^6^ cfu/ml *Pst* DC3000 Δ*avrPto* Δ*avrPtoB* or 10 mM MgCl_2_ (Table 1). Three biological replicates per infiltration were used and leaf samples were collected at 6 and 12 h after infiltration (hai), frozen in liquid N_2_ and stored at −80 °C until processed.

### RNA-Seq library preparation and analysis

Total RNA was isolated using TRIzol reagent (Life Technologies, NY, USA) and libraries prepared as described previously^18^. Barcoded libraries were multiplexed by 8–15 in each lane and sequenced on an Illumina HiSeq 2000 equipment with 45–50 bp single-end read mode. Sequence reads generated in this work have been deposited in the NCBI sequence read archive (SRA) under accession number SRP093524. Analysis of the RNA-seq data was performed as described previously^18^. Processed data generated in this work are available from the Tomato Functional Genomics Database (Tomato Functional Genomics Database [ http://ted.bti.cornell.edu/]).

### Selection of the reference genes using RNA-seq data and primer design

Expression data generated in this work, along with those generated in previous RNA-seq experiments^17^,^18^ shown in Supplementary Table S1, were used for the selection of the most stably expressed genes across all the treatments. Nine genes with lower variation coefficient (VC), calculated as the ratio between the standard deviation and the average of each gene expression (RPKM, reads per kilobase of transcript per million mapped reads) across all the treatments and biological replicates, were selected (Supplementary Table S2). Additionally, two traditional reference genes used in tomato (*GADPH* and *EF1α*) and *PHD*, the most stably expressed tomato gene identified in a previous report using *Xcv* infected tomato plants^38^ were included for analysis.

The nucleotide sequence of each gene was downloaded from the Sol Genomics webpage^47^ and primers were designed using the PrimerQuest tool (Integrated DNA Technologies). Primer efficiencies were checked by RT-qPCR using different cDNA dilutions (Supplementary Table S3). Dissociation curves were performed to show amplification specificity (Supplementary Fig. S2).

### RNA isolation and cDNA preparation

Total RNA was isolated using the Tri-Reagent (Sigma Aldrich) following the manufacturer’s instructions. RNA integrity was assayed by 1% agarose gel electrophoresis. Total RNA (8 μg) was processed with RQ1 RNase-free DNase (Promega) for 60 minutes at 37 °C to eliminate potential DNA contamination and then purified using a chloroform:octanol mix (24:1). RNA concentration and purity was determined using a NanoDrop 2000 spectrophotometer (Thermo Scientific). 2.4 μg RNA was used to prepare cDNA using M-MLV reverse transcriptase (Promega) with random primers according to the manufacturer’s instructions.

### RT-qPCR assay

RT-qPCR was performed as described previously^61^ in 96-well plates (Thermo Fisher Scientific) on the StepOnePlus system (Applied Biosystems). Primer sequences and characteristics are shown in Supplementary Table S3. The reaction mix was performed using: 5 μl of FastStart Universal SYBR Green Master (Rox) (Roche Life Sciences), 2 μl of 2 μM primer mix, 2 μl of a diluted 1:10 cDNA and water to complete a final volume of 10 μl. Cycling conditions were 95 °C for 10 minutes, and 40 cycles of 95 °C for 15 s, 60 °C for 1 min. All RT-qPCR experiments were performed using three biological and three technical replicates.

### Evaluation and validation of reference gene expression stability

Data obtained from the RT-qPCR experiments were analyzed using three statistical programs: geNorm^51^, NormFinder^52^ and BestKeeper^53^.

Expression of one PTI- (Solyc02g069960) and one ETI-specific gene (Solyc09g092500) was analyzed by RT-qPCR as explained above^18^. The data obtained was normalized using the two best and the worst reference genes and the relative expression was expressed as E^−ΔΔCq^, where E corresponds to the primer efficiency value.

## Additional Information

**How to cite this article**: Pombo, M. A. *et al*. Use of RNA-seq data to identify and validate RT-qPCR reference genes for studying the tomato-*Pseudomonas* pathosystem. *Sci. Rep.* **7**, 44905; doi: 10.1038/srep44905 (2017).

**Publisher's note:** Springer Nature remains neutral with regard to jurisdictional claims in published maps and institutional affiliations.

## Supplementary Material

Supplementary Information

Supplementary Table S1

Supplementary Table S2

## Figures and Tables

**Table 1:** Summary of the experiment performed for the RT-qPCR analysis.

Plant	Inoculum	Immune response	Concentration	Time points
Rio Grande (RG)-PtoR^a^	*Pseudomonas fluorescens* 55	PTI	10^8^ cfu/ml	6, 12 h
	*Pst* DC3000^b^	PTI/ETI	5 × 10^6^ cfu/ml	
	*Pst* DC3000 Δ*avrPto* Δ*avrPtoB*^c^	Disease	5 × 10^6^ cfu/ml	
	MgCl_2_	None	10 mM	

^a^Tomato Rio Grande-PtoR plants (Pto/Pto, Prf/Prf).

^b^*Pseudomonas syringae* pv. *tomato (Pst*) DC3000.

^c^*Pseudomonas syringae* pv. *tomato (Pst*) DC3000 mutant, lacking AvrPto and AvrPtoB effectors.

**Table 2:** Analysis of ten selected tomato reference genes using Bestkeeper algorithm.

Ranking	1	2	3	4	5	6	7	8	9	10
Gene name	*APX*	*ARD2*	*VIN3*	*TatC*	*KLC*	*GSN*	*3-IMDH*	*PHD*	*EF1α*	*GADPH*
Geo Mean [Cq]	22.33	21.01	26.59	21.78	24.46	24.89	22.42	19.40	15.14	18.81
Min [Cq]	21.54	20.26	25.86	20.90	23.76	24.34	21.87	18.53	14.42	17.09
Max [Cq]	23.27	21.64	27.41	22.38	25.18	25.63	23.33	20.51	16.67	22.46
**SD [±Cq]**	**0.34**	**0.28**	**0.33**	**0.26**	**0.31**	**0.31**	**0.32**	**0.36**	**0.45**	**1.23**
CV [% Cq]	1.51	1.32	1.23	1.18	1.25	1.25	1.44	1.85	2.96	6.51
Min [x-fold]	−1.73	−1.68	−1.66	−1.84	−1.62	−1.47	−1.47	−1.82	−1.64	−3.29
Max [x-fold]	1.92	1.55	1.76	1.51	1.65	1.66	1.88	2.16	2.89	12.53
**SD [±x-fold]**	**1.26**	**1.21**	**1.26**	**1.20**	**1.24**	**1.24**	**1.25**	**1.28**	**1.36**	**2.34**
**Coeff. of Corr. [r]**	**0.836**	**0.827**	**0.79**	**0.72**	**0.717**	**0.676**	**0.66**	**0.125**	**0.061**	**—**
*p*-value	0.001	0.001	0.001	0.001	0.001	0.001	0.001	0.561	0.774	—

[Cq], quantification cycle; Geo Mean [Cq], geometric mean of Cq; Min and Max [Cq], the extreme values of Cq; SD [Cq], standard deviation of Cq; CV [%Cq], coefficient of variance expressed as a percentage on the Cq level; Min [x-fold] and Max [x-fold], the extreme values of expression levels expressed as an absolute x-fold over- or under-regulation coefficient; SD [±x-fold], standard deviation of the absolute regulation coefficients, Coeff. of Corr [r], coefficient of correlation between each candidate and the BestKeeper index.

**Table 3:** Gene stability ranking established by the combination of geNorm, NormFinder and BestKeeper results.

Global ranking	Genes	geNorm	NormFinder	BestKeeper	Mean
1	*ARD2*	1	2	2	1.67
2	*VIN3*	1	1	3	1.67
3	*KLC*	2	3	5	3.33
4	*GSN*	4	4	6	4.67
5	*APX*	7	6	1	4.67
6	*TatC*	6	5	4	5.00
7	*Tspan*	3	8	ND	5.50
8	*Ube2N*	5	9	ND	7.00
9	*3-IMDH*	8	7	7	7.33
10	*PHD*	9	10	8	9.00
11	*EF1a*	10	11	9	10.00
12	*GADPH*	11	12	10	11.00

*ND*, Not determined.
