# Supplementary material for: Use of RNA-seq data to identify and validate RT-qPCR reference genes for studying the tomato-Pseudomonas pathosystem
Source: Sci Rep. 2017 Mar 20;7:44905. doi: 10.1038/srep44905 (PMC5357963; doi:10.1038/srep44905)
Supplement: Supplementary Information [file srep44905-s1.pdf]

Manuscript title

Use of RNA-seq data to identify and validate RT-qPCR reference genes for studying the tomato-*Pseudomonas* pathosystem

List of authors:

1. Marina A. Pombo
2. Yi Zheng
3. Zhangjun Fei
4. Gregory B. Martin
5. Hernan G. Rosli

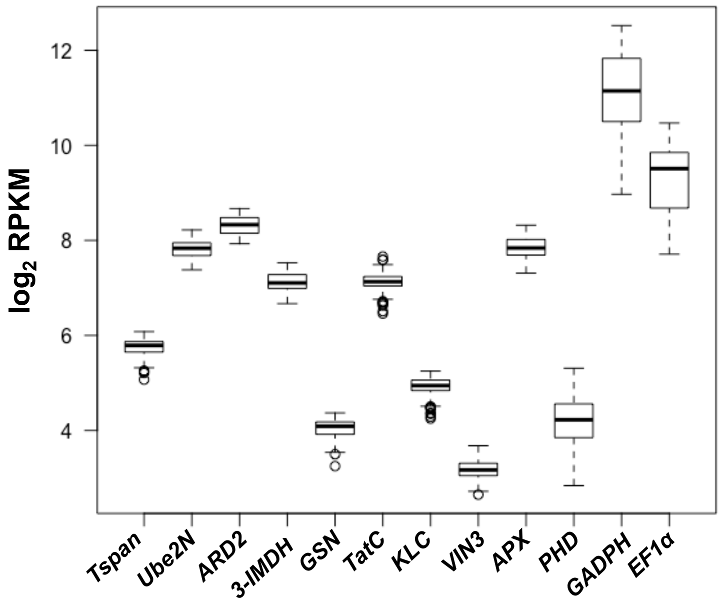

Supplementary Fig. S1: RNA-seq expression level (RPKM) of candidate and classical reference genes from tomato. Box and whisker plot graph showing $\log_2$ RPKM values of each selected gene in all the samples analyzed (n=110). Black lines and boxes represent the medians and 25/75 percentiles, respectively. Whisker caps represent the minimum and maximum values. **O**, indicate outliers.

Supplementary Table S3: Selected candidate gene primer characteristics

Gene	Transcript ID	Primer sequence	Amplicon length (bp)	Tm (°C)	Amplification efficiency (%)
Tspan	Solyc08g077220	TCTTATTGGCCCTCGTAGT	99	55.2	89
		GTGGCTGTCGGATTTGTT		53.9	
Ube2N	Solyc07g062570	TGGATAGCACCCTCCAAA	144	53.9	117
		CCCACAGATCTGAATCACTTAC		60.3	
ARD2	Solyc01g104170	TGTTCATCAGTGTGCTAGTG	99	56.4	99
		GCTGTCCTTCCTTCTGAATC		58.4	
3-IMDH	Solyc03g005730	CCTCAGCAGACTGAAAGAAA	106	56.4	95
		GGCTCACATCAGGCTTATC		57.3	
GSN	Solyc02g021420	GCCATGTAGTGCGTGTAAT	95	55.2	107
		CTCTGTCACTGCCACATTAG		58.4	
TatC	Solyc05g008530	CTAGGTCCAGGAGAGTTCTT	109	58.4	99.9
		CAGGAAGAACGAAGGCTATG		58.4	
KLC	Solyc00g082150	GCATTTGGCTTCAGAAGTTG	114	56.4	106
		CAACCAATAAGCTTCACCAG		56.4	
VIN3	Solyc07g018270	ACTGGTTTGCCTGTGAAG	114	53.9	98.7
		CTCAGAGAGAAGGGCATCTA		58.4	
APX	Solyc01g111510	CTCATTGTCGATCGGTTCTC	78	58.4	91.6
		CGACGAGCTTTCTCGATTT		55.2	
PHD[1]	Solyc06g051420	GGGATGGGATGGAGCGTAGAGA	279	65.9	87.3
		CATCACTCTCCTCTTGCAGCCT		64.0	
GADPH[1]	Solyc04g009030	CTGCTCTCTCAGTAGCCAACAC	156	64.0	83.3
		CTTCCTCCAATAGCAGAGGTTT		60.3	
EF1α[2]	Solyc06g005060	TCCAAAGATGGTCAGACCCGTGAA	119	67.6	86.2
		ATACCTAGCCTTGGAGTACTTGGG		59.8	
ETI-specific gene[2]	Solyc09g092500	TTGGACAGATCAAGGGACTAATG	95	54.3	99
		CACTCTCAACCACACCATCTT		54.7	
PTI-specific gene[2]	Solyc02g069960	AGCCAACAAAGCTCAGGAA	100	54.6	100
		CATCCCAGTTGCCATGTTCTA		54.9	

Primer sequence obtained from:

1 Muller, O. A. *et al.* Genome-wide identification and validation of reference genes in infected tomato leaves for quantitative RT-PCR analyses. *PLoS ONE* **10**, e0136499, doi:10.1371/journal.pone.0136499 (2015).

2 Pombo, M. A. *et al*. Transcriptomic analysis reveals tomato genes whose expression is induced specifically during effector-triggered immunity and identifies the Epk1 protein kinase which is required for the host response to three bacterial effector proteins. *Genome Biol* **15**, 492, doi:10.1186/s13059-014-0492-1 (2014).

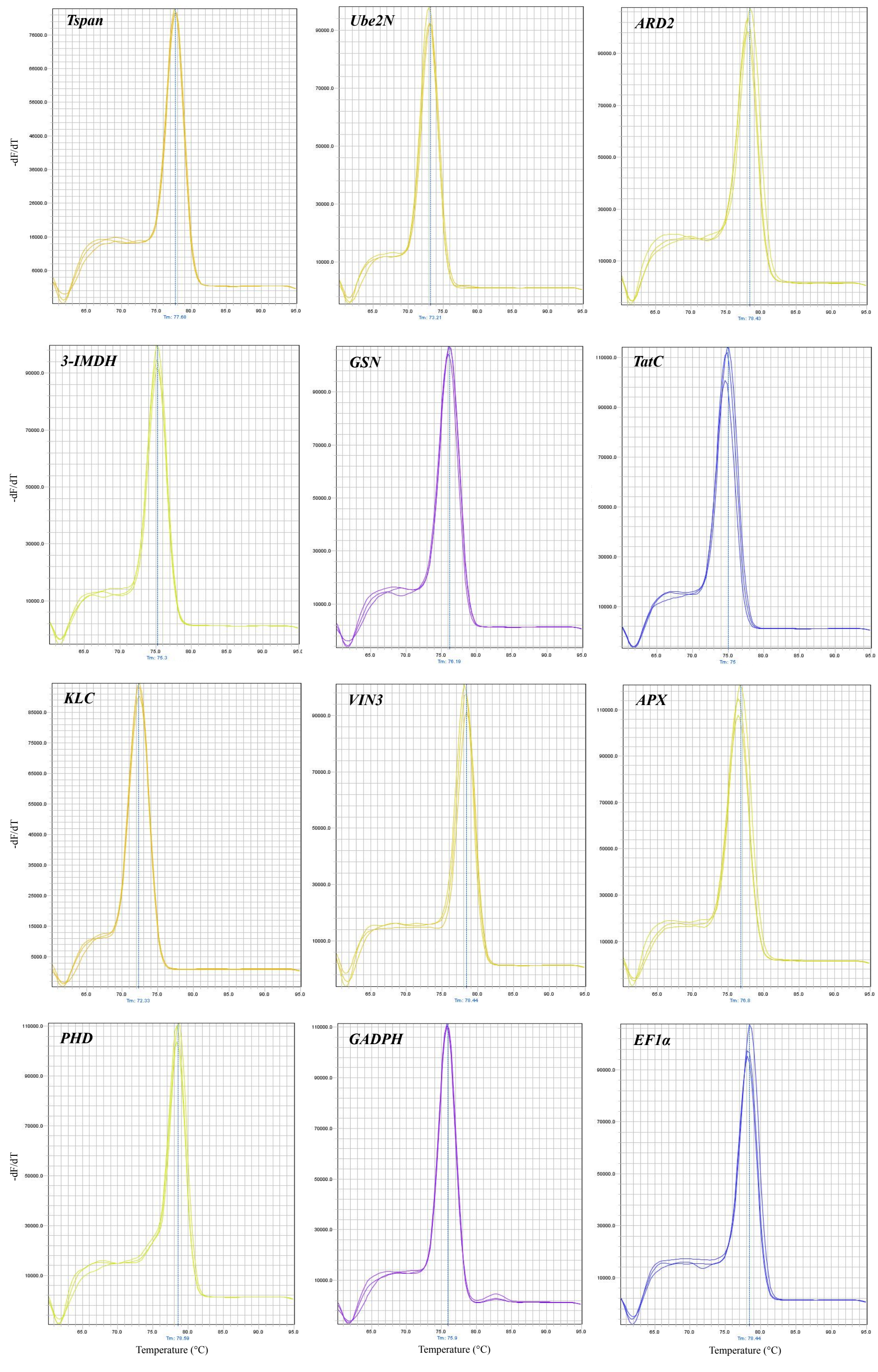

Supplementary Fig. S2: Validation of primer pairs of tomato candidate reference genes for RT-qPCR experiments. PCR amplification specificity was measured by the presence of unique amplicons using melting curve analysis.

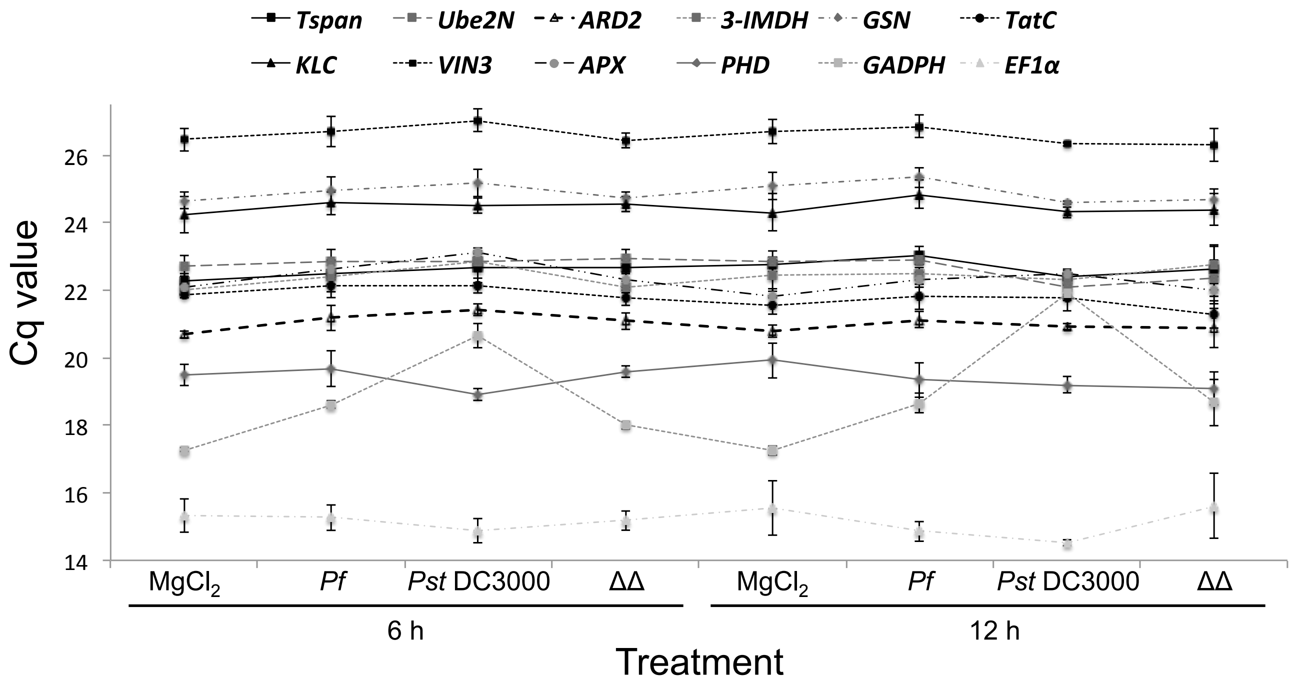

Supplementary Fig. S3: Expression pattern of selected reference genes along all the treatments performed for validation. Tomato leaves were syringe-infiltrated with 10 mM $MgCl_2$ ($MgCl_2$), 10^8 cfu/mL *Pseudomonas fluorescens* 55 (*Pf*), 5 x 10^6 cfu/mL *Pseudomonas syringae* pv. *tomato* DC3000 (*Pst* DC3000) or 5 x10^6 cfu/mL *Pst* DC3000 Δ*avrPto* Δ*avrPtoB* (ΔΔ). Samples were taken at 6 and 12 h after infiltration. Expression of reference genes was determined by RT-qPCR. Symbols indicate the average Cq of three biological replicates with three technical replicates and error bars represent standard deviation.
